# VMP35 MNC, a novel iron-free supplement, enhances cytoprotection against anemia in human subjects: a novel hypothesis

**DOI:** 10.29219/fnr.v63.3410

**Published:** 2019-05-09

**Authors:** Jean-Ronel Corbier, Bernard William Downs, Steve Kushner, Ted Aloisio, Debasis Bagchi, Manashi Bagchi

**Affiliations:** 1 Brain Restoration Clinic, A Division of Integra Wellness Center, Indian Land, SC, USA; 2 Victory Nutrition International, Inc., Department of R&D, Lederach, PA, USA; 3 ALM R&D, Oldsmar, FL, USA; 4 Veritas Health Inc., Woodbridge, ON, Canada; 5 Department of Pharmacological & Pharmaceutical Sciences, University of Houston college of Pharmacy, Houston, TX, USA; 6 Dr. Herbs LLC, R&D, Concord, CA, USA

**Keywords:** VMP35, a liquid multi-nutrient complex, anemia, human subjects, live blood cell imaging, morphology, hematology, rheology, safety

## Abstract

**Background:**

The American Society of Hematology reported that according to the National Heart, Lung, and Blood Institute (NHLBI) anemia is the most common blood disorder, which affects more than 3 million Americans, while the Global Burden of Disease 2016 (GBD 2016) reported that iron deficiency anemia (IDA) is the leading cause of anemia, which affects 1.93 billion people worldwide. Anemia is intricately linked to chronic inflammation, chronic kidney disease, gastrointestinal and gynecological malignancies, and autoimmune disorders. Hemorrhagic anemia results in substantial loss of blood, which causes significant alterations in all blood parameters, including reduced iron. The other type of anemia is chronic anemia syndrome (CAS), which is a constellation of disorders and chronic inflammatory events caused by an increasing anaerobic/acidic environment (promoting the growth of anaerobic organisms), inducing a defensive expenditure of alkalinizing buffers in hemoglobin (i.e. histidine), to prevent a dangerous lowering of blood pH. In this process, iron is cleaved from heme groups and transferred out of blood circulation into other organs, like the liver, appearing to be IDA, where excessive accumulation can lead to hemochromatosis, also known as ‘iron overload anemia’.

**Design:**

A pilot clinical study was conducted in 38 subjects (men = 10; women = 28; age = 22–82 years) to evaluate the rate of absorption and effects on blood of VMP35 multi-nutrient complex (MNC), a non-iron containing liquid nutraceutical supplement. Subjects consumed either placebo or VMP35 (30 mL) over a period of 0, 5, or 30 min.

**Methods:**

Changes in peripheral blood smears from 38 subjects were observed using live blood cell imaging (LBCI) with phase contrast microscopy. Adverse events were rigorously monitored.

**Results:**

VMP35 caused positive changes in the blood, including morphological, hematological (including restoration of hemoglobin), and rheological changes following 5 min of administration, which were sustained for at least 30 min.

**Conclusion:**

Overall, the non-iron containing VMP35 can induce improvements in blood properties and potential benefits for subjects even with compromised digestive systems. No adverse events were reported. Further research studies are in progress to explore the mechanistic insight.

## Popular scientific summary

- Anemia is the most common blood disorder which affects 1.93 billion people worldwide, and is associated with chronic kidney disease, gastrointestinal and gynecological malignancies, and autoimmune disorders.
- Our team hypothesized a term “chronic anemia syndrome (CAS)” which demonstrates the progressive inability of the human body to effectively use cellular oxygen. This in turn induces progressive acidemia in the blood supply and causes a metabolic shift toward cellular anaerobic glycolysis and a compensatory expenditure of alkalinizing histidine molecules from the heme protein of deconjugated hemoglobin, consequently releasing iron in the process.
- This clinical investigation assessed the efficacy of novel patent-pending VMP35 multi-nutrient complex, a non-iron containing liquid nutraceutical supplement, in 38 male and female volunteers (age: 22-82) on anemia and blood properties. Live blood cell imaging was performed using phase contrast microscopy.
- VMP35 induced significant improvements in blood health parameters following 5- and 30 min of oral intake.
- VMP35 intake consistently restored RBC hemoglobin and caused marked improvements in blood properties as well as improved digestion and overall health.

Anemia is a health deteriorating condition of having a lower than normal red blood cell (RBC) count, or a condition where RBCs do not have adequate hemoglobin (Hb) to effectively carry sufficient oxygen to the body’s vital organs and tissues (1–5). Thus, deprivation of oxygen-rich RBCs is the prime characteristic of anemia, which may occur in both men and women at any age group for a temporary or a longer period (3–8). Also, anemic condition can be mild to severe (2–4). However, women are more prone to anemia during pregnancy and childbearing age (5). The signs and symptoms of anemia include fatigue or tiredness, weakness, exhaustion, pale or yellowish skin, irregular heartbeats, shortness of breath, dizziness or lightheadedness, chest pain, cold hands and feet, and headache (5, 9–11).

Presently, five main causes of anemia are reported: (1) blood loss due to excessive menstrual bleeding or bleeding in the digestive or urinary tract, surgery, trauma, and cancer, (2) hemorrhagic anemia due to substantial loss of blood, leading to significant alterations in the blood parameters including reduced iron, (3) lack of ability to produce RBCs, (4) hereditary conditions of compromised rate of RBC production, and (5) chemotherapy or radiation therapy (8–11).

There are four major iron-containing proteins available in the human body: (1) mononuclear proteins, (2) diiron-carboxylate proteins, (3) iron-sulfur proteins, and (4) heme proteins. Heme proteins are most abundant in the human body. It is important to note that Hb, located in erythrocytes or RBCs, contains approximately 50% of the total body iron. Thus, Hb plays a key role in anemia. Erythropoiesis, a process for the production of erythrocytes, is regulated by three prime factors: (1) appropriate oxygenation of tissues and organs, (2) turnover of erythrocytes, and (3) loss of erythrocytes due to hemorrhage (9–14).

Erythrocytes and their precursors, erythroblasts, need a requisite amount of functional iron and oxygen for the production of Hb and heme (1–4). It is well known that iron is centrally located in the Hb structure and is essential for Hb functions (5, 6). It is well established that erythroblasts are nucleated cells found in the bone marrow and are the precursors of erythrocytes. Mono- and diferric transferrins, available in considerable amount in plasma, are the immediate sources of functional iron for erythroblasts (7–9). Generally, anemia is associated with low iron function in available transferrin (5). Iron is available from three major sources: (1) gut, (2) macrophages, and (3) hepatic tissues, a storage of ferritin iron (1–6). Generally, iron storage declines or is lost before anemia develops. Hence, dietary and rejuvenated iron has been considered the primary necessity for erythrocyte production (1–3, 9–13).

When there is no hemorrhage or diseased conditions, tissue oxygenation and erythrocyte production are stable during adulthood (5–7, 12). During hemorrhage, oxygen deprivation and iron availability dramatically decline, which may lead to anemia and fatigue (9–11). Moreover, anemia is intricately linked to chronic inflammation, chronic kidney disease, gastrointestinal and gynecological malignancies, and autoimmune disorders (1–3, 7–10).

Chronic anemia syndrome (CAS), an umbrella term coined by our team which covers all other forms of iron deficiency anemia (IDA) not due to genetic or hemorrhagic conditions. This is a constellation of chronic inflammatory disorders induced by an increasing anaerobic/acidic environment and thereby promotes the growth of anaerobic organisms (11). In other words, CAS is characterized by a deficiency of primary intracellular alkalinizing ion buffers inducing a defensive expenditure of secondary alkaline buffers from Hb (i.e. histidine), to prevent a remarkable lowering of blood pH. It is well known that a heme group is attached to four histidines, while the iron atom in heme binds to the four nitrogens in the center of the protoporphyrin ring (14, 15). Under this condition, iron is cleaved from the heme protein to release histidine. Subsequently, iron is taken out of circulation and stored in the hepatic storage depots and other tissues, which lead to excessive accumulation of iron in the organs and ultimately lead to hemochromatosis, a pathophysiological condition known as “iron overload anemia” (IDA) (16). In severe hemochromatosis, iron saturation in the organs results in a downregulation or cessation of deposition in those organs that causes excessive accumulation in blood plasma (16, 17). RBCs are unable to restore Hb status due to the excess expenditure of histidine from heme proteins to maintain ideal alkaline blood pH properties and oxygen utilization (14–17).

As stated before, we hypothesized a term ‘chronic anemia syndrome (CAS)’ which demonstrates the progressive inability of the human body to effectively use cellular oxygen, which in turn induces a progressive acidemia in the blood supply as evidenced by an increasing hypochromic state in the RBCs due to a significant loss of Hb. This cascade of events forces a metabolic shift toward cellular anaerobic glycolysis, and a compensatory expenditure of alkalinizing histidine molecules from the heme protein of deconjugated Hb, which releases iron.

To test our hypothesis, we assessed the efficacy of oral administration of a novel and safe non-iron containing VMP35 multi-nutrient complex (MNC) encapsulated in a proprietary SK713 SLP phospholipid Prodosome technology on anemia and blood properties (i.e. Hb) in human volunteers (18). This formulation technology is biodegradable and biocompatible. The research was intricately designed to observe the rate of absorption of Prodosomed VMP35 ingredients by evaluating its effects on Hb (and neutrophils) of live human blood. Furthermore, we included a case study report conducted by an independent physician in Norwich, NY, USA.

## Materials and Methods

### Novel VMP35 MNC vitamin mineral phytonutrient nutraceutical formulation

This novel VMP35 MNC, a vitamin, mineral, and phytonutrient encapsulated liquid formulation, was prepared using a novel proprietary SK713 SLP multi-lamellar clustoidal non-GMO phospholipid Prodosome nutrient absorption/delivery technology in a state-of-the-art multi-step cGMP and NSF-certified manufacturing facility. Note that this developmental technology is biodegradable and biocompatible. The product manufacturing batch/lot number used in this clinical investigation was 13070-2 (manufacturing date: March 22, 2013).

A unique technology was conducted in a multi-step manufacturing process; in the first step, the production of SK713 SLP was performed using a minimum of 85% non-GMO phosphatidylcholine, which was impregnated and saturated using solar-dried electrolytes to ensure the availability of free ions, which will amplify the ionic properties of the multi-lamellar clustoidal phospholipid spheres. In the second step, a combination of research-driven structurally diverse antioxidants, multivitamins, micronutrients, minerals, and standardized botanical phytonutrients was carefully blended utilizing a progressive high-shear wet milling treatment to create a nano-emulsion. The final step involved a specific blending, impregnation, and encapsulation technology to achieve a novel multi-lamellar energetically enhanced clustoidal ‘Prodosomal’ liposome-type encapsulated supplement (Prodovite).

### Study participants and recommended dose

A total of 38 subjects (male = 10; female = 28; age = 22–82 years) were randomly recruited from medical health clinics during interviews in Woodbridge and Perth, ON, Canada (see Table 1). All subjects were advised to orally take 1 oz VMP35 and swirl in the mouth for 30 s and then ingest it. Blood samples were taken at 0, 5, and 30 min after orally taking the formulation.

**Table 1:** Study participants, age and health problems

Participant	Age (year)	Sex	Ethnicity	Health issues
10	37	F	Guyanese	None reported
11	45	M	Canadian	High blood pressure (HBP)
12	37	F	Canadian	None reported
13	43	F	Canadian	Poor digestion
14	70	F	Italian	Osteoarthritis, osteoporosis
15	24	M	Lebanese	None reported
16	22	F	Canadian	None reported
17	22	F	Canadian	None reported
18	61	M	Canadian	None reported
19	51	F	Canadian	None reported
20	37	M	Canadian	None reported
21	62	F	Canadian	Skin condition
22	54	F	Canadian	None reported
23	63	F	Canadian	Diabetes
24	58	F	Canadian	None reported
25	43	M	Canadian	Digestion problem
26	49	F	British	None reported
27	51	F	Canadian	None reported
28	24	F	Canadian	Attention deficit disorders (ADD)
29	61	F	British	Thyroid, severe pain
30	56	F	Canadian	None reported
31	60	F	British	None reported
32	58	F	British	Depression, thyroid, hormone
33	79	F	Canadian	HBP, diabetes, heart problems
34	35	F	Canadian	None reported
35	40	F	Canadian	None reported
36	57	F	Canadian	None reported
37	40	F	Canadian	Depression
38	30	M	Canadian	None reported
39	70	F	Canadian	None reported
40	44	M	Trinidadian	None reported
41	50	M	Italian	None reported
42	50	F	Canadian	Toxic exposure
43	71	M	British	Severe periodontal disease
44	74	F	Italian	HBP
45	82	M	Italian	Bladder cancer, chronic lymphocytic leukemia (CLL)
46	33	M	Canadian	Herpes
47	56	F	Italian	None reported

Live Blood Cell Imaging in this sample population demonstrated that even in none-reported subjects widespread immunological challenges are evidenced by unanimous and significant cell aggregation and poor blood cell rheology. A total of 38 subjects (27 female and 11 male subjects, aged between 22 and 82 years) participated in this clinical investigation.

### Ethical approvals

An institutional review board approval was obtained from the Path Foundation in New York, NY (#13-009 April 25, 2013) and all subjects signed an informed consent form.

### Study design

This observational, pilot, controlled one-way crossover study was designed to evaluate the effect of a non-iron containing VMP35 liquid MNC (treatment group) on blood oxygenation and hydration compared to the placebo group (control) at baseline, 5 min and 30 min post-treatment, respectively. This clinical evaluation investigated the rate of absorption as assessed by effects on various properties of live human blood induced by the orally consumed VMP35 liquid nutraceutical supplement. Changes in peripheral blood smears (PBSs) from baseline (0 min) were observed using live blood cell imaging (LBCI) with phase contrast microscopy at 5 min post-control intake, and at 5 and 30 min post-supplement intake.

### Live blood cell imaging

This was conducted by Veritas Health Inc (Woodbridge, ON, Canada) using an Olympus BX-30 light microscope equipped with a phase contrast condenser (Tokyo, Japan). A 150-W lightbox with fiber-optic cable assembly was used to highlight the specimen against a gray field and increase the range of intermediate shades. The lighting produces a high level of cell definition, clear morphology, and can distinguish features of some cell membranes. The lens configuration was 10X eyepiece and 100 X-oil immersion objective magnification to achieve approximately 1,000 X magnification. Oil immersion achieved finer resolution and brightness.

### Blood smear handling

PBS was obtained from the finger using a Bayer Single-Let Disposable Lancet 23G 2.25 mm sterile single-use lancing device (Whippany, NJ). Being careful not to squeeze the finger, a small amount of capillary blood was expressed due to capillary pump action, collected, and transferred directly onto microscope slides. The slides used were pre-cleaned using standard 1 inch × 3 inch sterile gauze pad with a thickness of 1 mm supplied by Electron Microscopy Sciences (Hatfield, PA). The slide was covered quickly and gently with a cover glass without pressure to protect blood cells from damage. The cover glass was pre-cleaned with 22 mm × 40 mm sterile gauze pad with 0.13 to 0.17 mm thickness supplied by Electron Microscopy Sciences (Hatfield, PA). The corners of the cover glass were tapped carefully to disperse surface tension and create an even layer for viewing. The slide was then transferred directly to the microscope for viewing. Evaluation of blood properties began in less than 30 s after the blood samples were collected. Consistent blood extraction and handling procedures were followed to avoid artifacts. A PBS was drawn to assess both the placebo and treatment samples over a period of 0, 5, and 30 min post-treatment.

### Adverse events

A large number of participants upon entry into the study were suffering from an array of chronic diseases, including anemia. Adverse events monitoring was strictly enforced.

### Case study report

An independent case study report was added with proper consent from the patient who had a stroke in early 2018. The patient took 2 to 3 oz of Prodovite VMP35 per day over a period of little over 6 months. The time-dependent improvement has been included in the next section.

## Results

The findings of the study were encouraging. Table 1 exhibits the LBCI in a total of 38 subjects (male = 11; female = 27; age = 22–82 years) who participated in this clinical investigation. Significant improvement in blood properties, including hemoglobinization, of the treatment group was observed following treatment with Prodosome encapsulated non-iron VMP35 MNC nutraceutical formulation. Details have been demonstrated as follows.

### Phase contrast microscopy

Control group

No changes were observed between the baseline and 5-min samples.

Treatment groups

Substantial differences were observed between the baseline and 5-min samples after Prodovite VMP35 supplementation. Group 1: Figs. 1a (baseline before water intake), 1b (5 min after water intake), and 1c (5 min after VMP35 intake) and Group 2: Figs. 2a (baseline before VMP35 supplementation) and 2b (5 min after VMP35 supplementation).

**Group 1:**
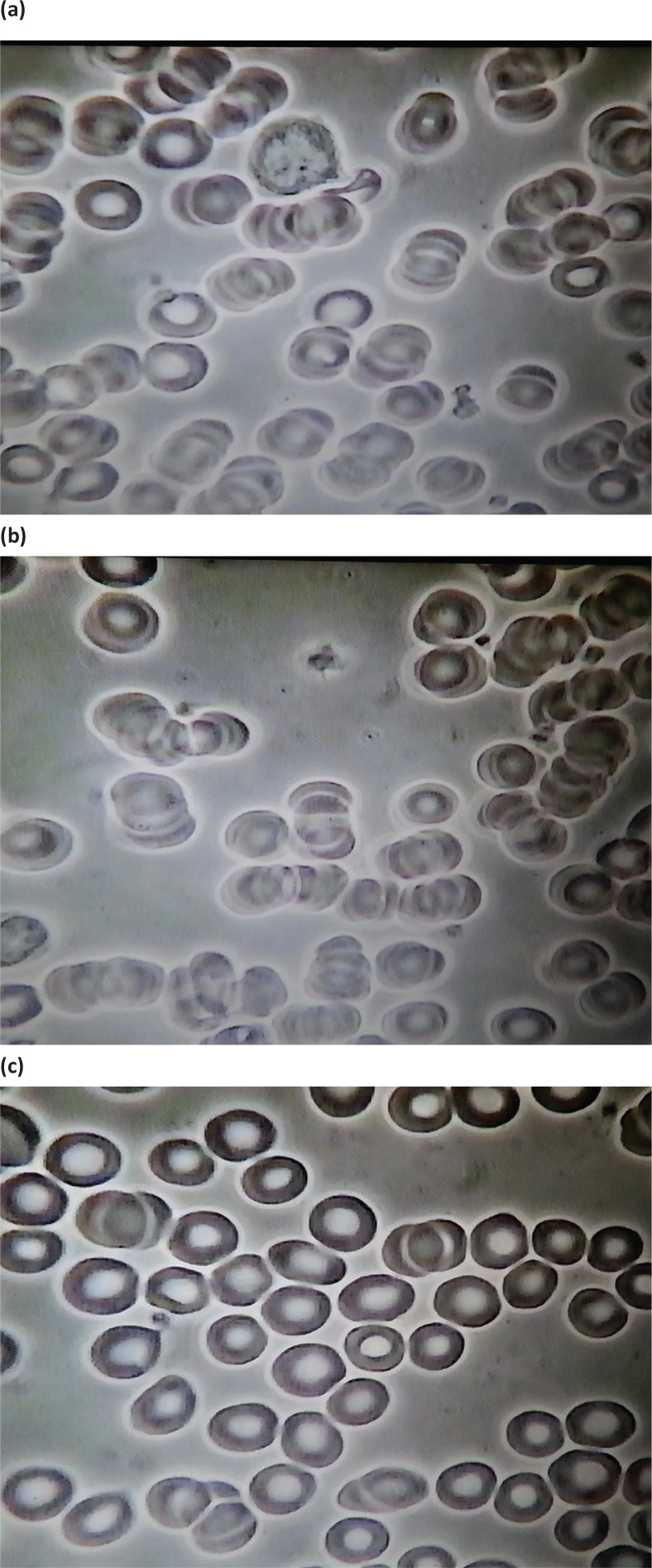
Baseline and 5 min after drinking water control and 5 min after VMP35 supplement. *Fig. 1a*. Live blood cell imaging of subject #41 1STILL1BW41 (baseline before water). *Fig. 1b*. Live blood cell imaging of subject #41 1STILL1AW41 (5 min after water). *Fig. 1c*. Live Blood cell imaging of subject #41 1STILL5AM41 (5 min after VMP35 supplementation).

**Group 2:**
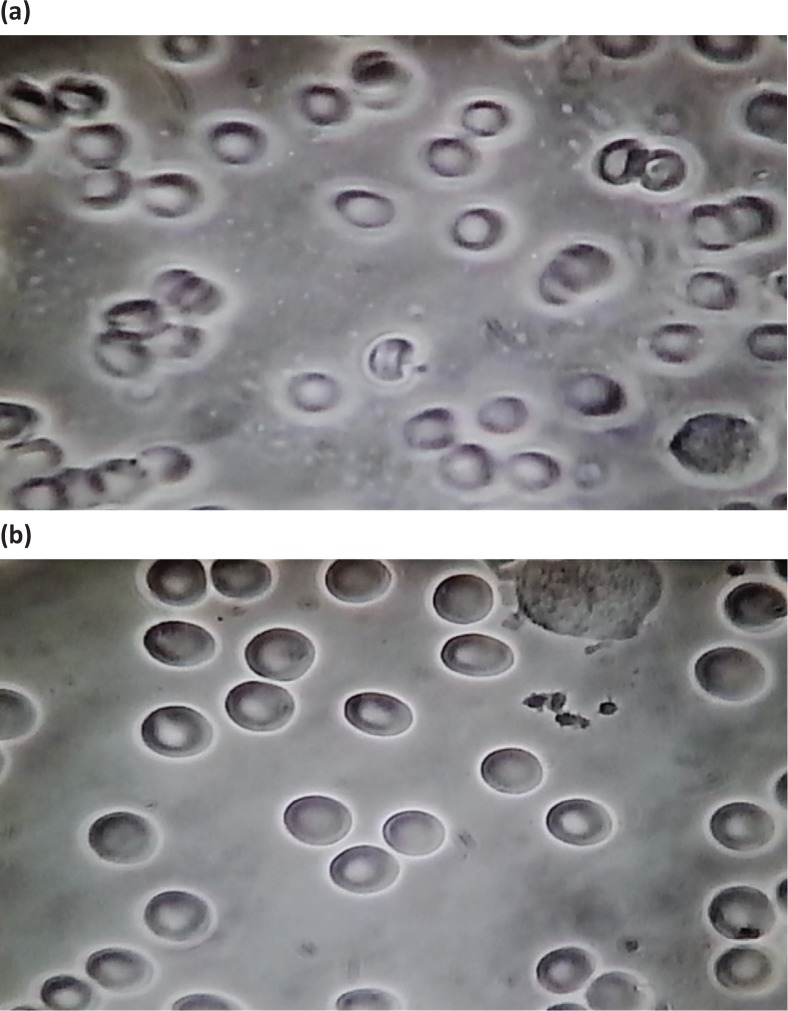
Baseline and 5 min after VMP35 supplement. *Fig. 2a*. live blood cell imaging of subject #17 2STILL3BM17 (baseline before VMP35 supplementation). *Fig. 2b*. Live blood cell imaging of subject #17 2STILL1AM17 (5 min after VMP35 supplementation).

30-min post-treatment group

Substantial differences were observed between the baseline, 5-, and 30-min post-active groups. Group 3: Figs. 3a (baseline before VMP35 supplementation), 3b (5 min after VMP35 supplementation), and 3c (30 min after VMP35 supplementation).

**Group 3:**
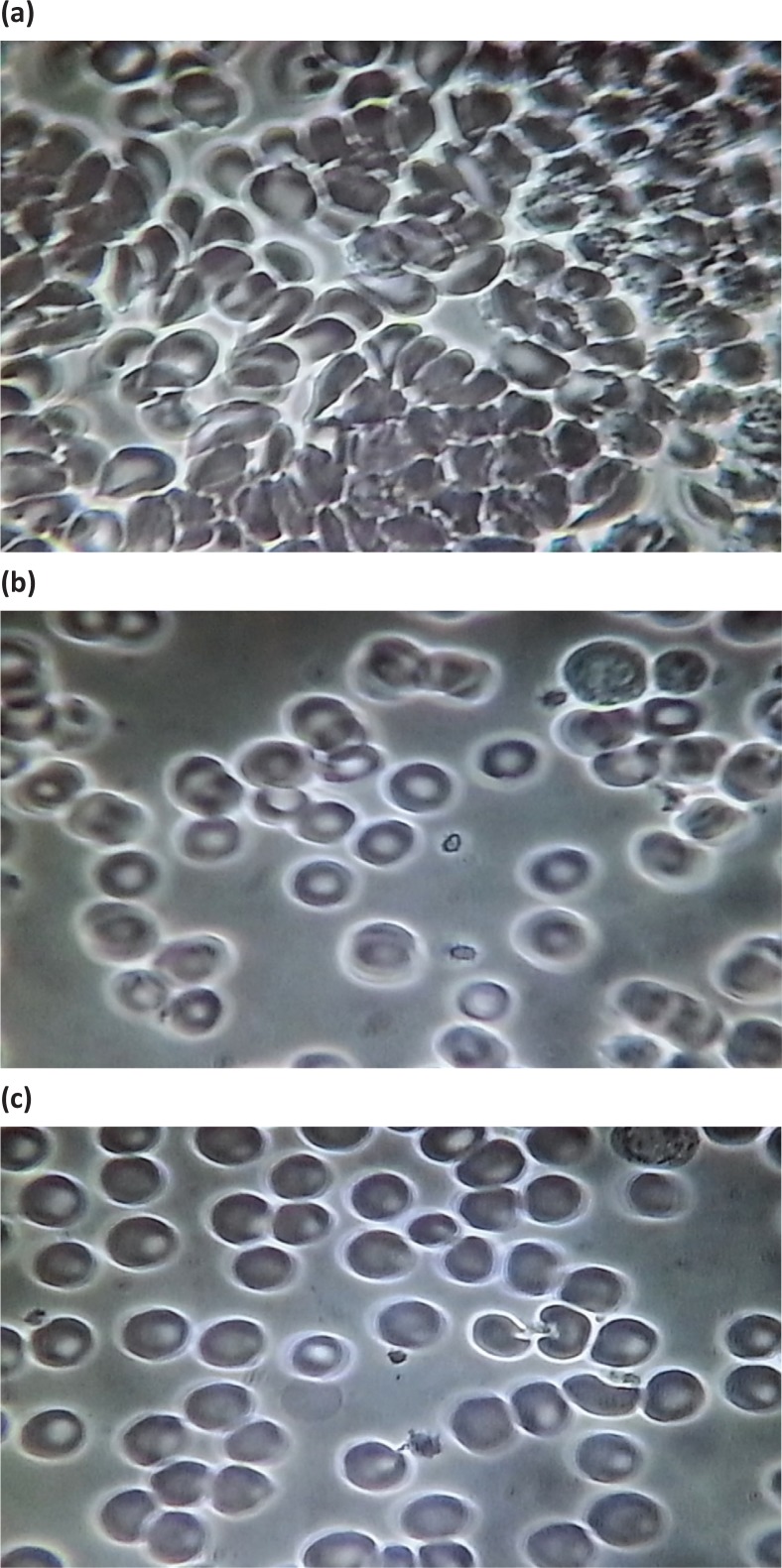
Baseline and 5 and 30 min after VMP35 supplement. *Fig. 3a*. Live blood cell imaging of subject #45 3STILL1BM45 (baseline before VMP35 supplementation). *Fig. 3b*. Live blood cell imaging of subject #45 3STILL3AM45 (5 min after VMP35 supplementation). *Fig. 3c*. Live blood cell imaging of subject #45 3STILL1THIRTY45 (30 min after VMP35 supplementation).

Following are the itemized observations in the baseline, post-supplement RBC improvements after 5 and 30 min, respectively.

Baseline

Observations in the control group and 5-min post-treatment samples included aggregation and immobility – a sludge effect; malformation, damage, and extensive hypochromic state (i.e. an oversized ‘donut hole’ evidencing reduced Hb). At baseline, extensive ‘debris’ in the plasma and ‘dwarfed’ white blood cells were also observed. A cross-section of ages and a variety of conditions were represented by the subjects so that the individual baseline PBS examples shown in the figures do not look similar.

### Post-supplement RBC infrastructural improvements after 5 min post-treatment

Post-supplementation RBC improvements after 5 min included a breakup of aggregation and splaying out of RBCs on the slide, demonstrating reduced viscosity, improved RBC spherical formation, and a progressive reduction (with time) of hypochromicity with RBCs refilling with Hb. Other post-supplementation observations include improved movement and ability to flow (rheology) of RBCs in plasma, evidence of improved hydration, reduced viscosity, and reduced surface tension (data not shown).

### Post-supplement RBC infrastructural improvements after 30 min post-treatment

Group 3 samples were evaluated at 5- and 30 min post-intake of the VMP35 and showed marked improvement in biomarkers from baseline. Post-Supplementation at 5- and 30-min demonstrated reduced or eliminated hypochromicity along with improved Hb concentration and a reduction in plasma debris. The plasma appeared cleaner, possibly due to a reduced quantity of sequestrants (of unknown origin), which are greater in number in the blood with greater cellular aggregation and reduced hydration (Fig. 3a–3c). Overall, improvements in the splayed arrangement, size, form, density, and distribution of RBCs following the intake of the VMP35 were observed.

Taken together, VMP35 caused dramatic morphological, hematological (including restoration of Hb and neutrophils), and rheological changes in the blood following 5 min of administration, which were sustained for at least 30 min. Improved blood rheology was observed by videographic assessment of live RBC movement on microscope slide, which is represented in still photograph by a significant reduction in RBC aggregation, improved RBC morphology, and distribution. Overall, VMP35 induced rapid improvements in blood properties, restored RBC Hb saturation and morphology, and improved neutrophil morphology within 5 min that were sustained for 30 min post-supplement intake. Following are the salient findings.

- RBC and blood rheology improvements were observed demonstrating that VMP35 was delivered and absorbed by sublingual trans-mucosa within 5 min.
- VMP35 exhibited a unique ability to initiate rapid onset of hematological changes in response to intervention.
- Changes were observed within 5 min of VMP35 administration, which were sustained for at least 30 min. Group 2 at 5 min and Group 3 at 5 and 30 min support the validity of the observations. No effect was observed in the control group. Prompt, sustained, and progressive results were observed in the treatment groups
- Overall, VMP35 exerted a rapid positive response on morphological, hematological, and rheological properties of the blood.

### Adverse events monitoring

No significant adverse events were observed. A significant number of subjects were already suffering from an array of chronic disorders during entry into the study. However, no treatment-related adverse events were noted in either placebo or treatment groups.

## Case study report in an anemic stroke patient

A recent *Case Study* reported the effect of oral supplementation of Prodovite VMP35 over a period of slightly more than six consecutive months in a subject in Norwich, NY, USA. Necessary permission to include this report in the study was obtained.

Physicians involved: Dr Piotr Sadej, MD; Dr Sundar Jayaraman, MD; and Dr Karen R Banks-Lindner, DO, FLLC. The detailed Doctor Case Report was provided with permission by Mr David J. Evans.

Subject: A 56-year-old Caucasian male stroke (cerebral infarction) patient suffering from anemia as demonstrated from his low Hb level of 2.8 gm/dL, which is far below the normal Hb level in a normal human subject.

In the following, the case history, treatment profile and regimen, and consequential events that occurred over a period from January to December 2018 are presented.

*January 5, 2018*: The patient had a multi-planar multi-sequence MRI of the brain (on a 1.5 T MRI system) as a follow-up of a CT done the previous day owing to a stroke (right-sided weakness, and hypertension). MRI revealed a left para-median distribution infarct specifically involving the pons and the medulla. Chronic lacunar infarcts and possible subacute white matter infarcts were also seen. These chronic changes were seen in the setting of white matter disease that could relate to microangiopathy. Multiple tiny foci of iron-containing hemosiderin evident in bilateral thalami, basal ganglia, brainstem, cortical/subcortical regions, and cerebellum were noted.

*CBC 5 weeks (mid-February 2018) post-stroke*

RBCs: 4.42 (normal range being 4.0 to 5.8)

Hemoglobin 2.8 gm/dL (normal range being 13.0 to 18.0) indicates anemia

Hematocrit: 38.1 (normal range being 37.0 to 52.0) – low normal evidence of anemia

Platelet count: 172 (normal range being 150 to 450) – low normal can indicate a trend toward an anemic condition

RDW 15.8 (normal range being 11.5 to 14.0) – a higher number indicates probable IDA

Low Hb level continued for approximately a little over 3 months when the subject started consuming 2 to 3 oz/day of Prodovite VMP35 from the middle of May 2018.

*May 2018 test results*

- Creatinine: 1.5 mg/dL (normal range being 0.5 to 1.2) indicates kidney challenges
- Glucose: 105 mg/dL (normal high limit being 99) – blood sugar is high

*August 2018 test results*

- RBCs 4.59 (4.0 to 5.8)
- Hemoglobin 14.4 gm/dL (normal range being 13.0 to 18.0)
- Hematocrit 42.3 (normal range being 37.0 to 52.0)
- Platelet count: not available
- RDW 12.6 (normal range being 11.5 to 14.0)

Improvement was observed in RBCs with significant improvements exhibited in other parameters.

*November 2018 test results*

- RBCs: 5.01 (normal range being 4.0 to 5.8)
- Hemoglobin 15.6 gm/dL (normal range being 13.0 to 18.0)
- Hematocrit: 45.9 (normal range being 37.0 to 52.0)
- Platelet count: 202 (normal range being 150 to 450)
- RDW 12.9 (normal range being 11.5 to 14.0)

These data demonstrate significant improvement in all parameters.

Consequential physician’s evaluation and findings on December 6, 2018

A follow-up brain MRI was conducted due to the history of cerebral infarction and the patient’s right hemiparesis and gait disturbance. This study was compared with the previous brain MRI from January 5, 2018. No acute infarction was observed. Sequela of previous infarction of the lower pons and upper medulla were seen (pontine and cerebellar encephalomalacia) along with evidence of previous hypertensive microhemorrhages (microscopic changes in the cerebral white matter).

Along with the above MRI, a magnetic resonance angiogram (MRA) was done at the same time, with the following findings: there was no occlusion or hemodynamically significant stenosis of major intracranial arteries.

### Overall conclusion

This report is very encouraging which demonstrates that non-iron containing Prodovite VMP35 has the ability to significantly improve hematological characteristics in the subject.

## Discussion

Anemia is an alarming health condition of having lower than normal RBC counts or when RBCs do not have adequate Hb and/or enough oxygen-rich blood. WHO reported in 2008 (19) that anemia affects 24.8% of world’s population, including 47.4% preschoolers, 25.4% school-age children, 41.8% pregnant women, 30.2% non-pregnant women, 12.7% men, and 23.9% of elderly population (19). Kassenbaum et al. (1, 3) estimated that in 2010 the global prevalence of anemia was 32.9% and extrapolated in terms of years lived with a disability, the total cumulative amount of time that people were disabled equaled 68.4 million years (20, 21). Anemia is closely associated with and a precursor of diverse disease pathologies, including chronic inflammation, chronic kidney disease, gastrointestinal and gynecological malignancies, and autoimmune disorders, while most of these disorders are preceded by an array of dysregulated metabolic events (22). According to the American Society of Hematology and National Heart, Lung, and Blood Institute (NHLBI), anemia affects more than 3 million Americans (23).

As indicated earlier, anemia is prevalent in diverse disease pathologies, most of which are preceded and characterized by an increase in anaerobic metabolic events (24–27). Optimal health is the result of the body’s ability to successfully maintain the ideal biological environment for optimal cellular functioning. Aerobic cellular events are important for human life (27–29). Maintaining a highly efficient pH buffering system, a pH between 7.35 and 7.45, is ideal for maintaining optimal oxygenation of the blood in addition to many other biological processes (30, 31). Optimizing the ideal pH in the blood is the result of a homeostasis of acid and alkaline pH buffers. A pH of the blood below 7.35 is acidemia, while a pH above 7.45 is alkalemia, and a pH of 7.40 is ideal (29–33). Due to the importance of sustaining a pH level in the narrow specified range, the human body exerts a compensatory mechanism to induce a homeostatic counterbalance (30–33).

Now let us revisit the array of chronic diseases, which can also be characterized by an increased inability to effectively utilize oxygen, resulting in anaerobic metabolism and lactate accumulation (34). Impairment of oxidative pathways during lactate production results in a net gain of H^+^ with increasing cellular acid burden, thereby decreasing the blood pH (34).

As discussed earlier, the etiology of CAS is a progressive inability of the human body to effectively use cellular oxygen, inducing progressive acidemia, a metabolic shift toward cellular anaerobic glycolysis, and a compensatory expenditure of alkalinizing histidine molecules from the heme protein of deconjugated Hb, which releases iron. Iron is taken out of circulation and accumulates in the liver, bone marrow, and other organs, which appears to be IDA but can result in dangerous accumulations of iron. In addition to iron accumulating in certain organs, the consequences of an increasing anaerobic/acidic environment, especially in the blood, can manifest in a number of ways, in various tissues, and produce a wide range of symptoms and pathological manifestations including chronic and acute infections, flukes, vaso-occlusive incidences, CVD, strokes, kidney disease, cancers, diabetes, tuberculosis, HIV, endocarditis, osteomyelitis, and inflammatory bowel diseases such as Crohn’s disease, to name a few, all of which are preceded by CAS.

Let us discuss the intricate aspects of CAS and explore its association with Hb, which is usually checked in a complete blood count (CBC). Structurally, Hb is composed of four chains – two α-globulin and two β-globulin chains – and each chain is known as heme that contains iron and is responsible for transporting oxygen in the bloodstream (35, 36). Basically, Hb is a protein molecule in RBCs that transports oxygen from the lungs and delivers it to the peripheral tissues to maintain the viability of cells and returns CO_2_ from the organs and tissues back to the lungs. An upsurge of CO_2_ acts as an acidifying buffer. CO_2_ interacts with water to form carbonic acid and results in a decrease of blood pH. Also, the pigment in Hb is responsible for its red color. The normal average range of Hb lies between 14 and 18 g/dL for an adult male, and 12–16 g/dL for an adult female (35, 36). Abnormal levels or deformation of Hb can lead to serious diseases or consequences, while low Hb level is referred to as anemia. Hb is responsible for the shape of the RBCs, which looks like a donut with a thin center, instead of a hole. However, for dysfunctional or deformation of Hb, the shape becomes abnormal, which is predominant during anemia (35) (Figs. 1a–c, 2a–c, 3b and c).

Anemia has been demonstrated to be caused by factors that interfere either with the compromised Hb level or the absence of adequate RBCs or oxygen deprivation. There are six underlying causes: (1) loss of RBCs due to bleeding, as in hemorrhagic anemia, (2) lack of production of RBCs in the bone marrow, (3) hemolysis or breakdown or deformation of RBCs in the blood stream, (4) nutritional deficiency or inadequate intake of iron, folic acid, or vitamin B12, (5) kidney disease and (6) genetic predisposition (35, 36).

Compromised Hb proteins ultimately release their load of oxygen (36–38). In conjunction, increased acid burden in the blood can be characterized by an increase in the CO_2_ load in the blood gas, and this effect induces a secondary role in ionizing minerals in the kidney. This is a reason why anaerobic disorders promote kidney problems (12, 13).

Hb is divided into two types:(1) oxyhemoglobin, the red oxygen-carrying form, and (2) deoxyhemoglobin, the blue/purple deoxygenated or reduced form. Hb is also expected to perform an important task as a pH buffering agent to regulate and optimize cellular oxygen utilization. This role is primarily dependent on Hb’s histidine content of its heme groups (14). Under pH regulatory distress, as in increasing acid burden, and a significant increase in alkalinizing buffers, heme groups are broken down to release histidine (38–41). Iron is released simultaneously with the histidine and is rapidly sent to other organs such as the liver, bone marrow, heart, pancreas, intestinal mucosa, etc. During the transport, iron remains in the blood for a very short time. However, in more extreme cases, the iron accumulates in vital organs, which contributes to the development of several diseases including hepatic cirrhosis, hepatocarcinoma, cardiac cirrhosis, diabetes mellitus, osteoarthritis, osteoporosis, and non-genetic secondary hemochromatosis also known as ‘iron-overload anemia’, as blood iron levels can be initially low with accumulated tissue levels being too high (15). This is a major reason why blood tests can be inaccurate in determining IDA. However, prolonged accumulation of iron in the organs can lead to critical saturation in the tissues and iron overload with a downregulation of receptor sensitivity and once again elevate blood iron levels. Furthermore, increased saturation of transferrin and high iron in plasma (i.e. extracellular concentrations) are hallmarks of hemochromatosis forms. Tests for evaluating this condition include serum transferrin saturation, serum ferritin, liver function tests, MRI, and liver tissue biopsy (16, 17). It is important to note that it is not uncommon that elevations in one or all these blood tests for iron can be found in other disorders as well owing to the prevalence of the anaerobic/acidic bio-environment underlying chronic disease pathologies.

This research team hypothesized that the most effective remedy for such CAS disorders would be to replenish appropriate nutrient resources other than iron, thereby reanimating primary pH buffering capabilities and restoring healthy Hb properties, functions, and effective oxygen utilization. The benefit of this safe and routine practice would significantly reduce oxidative stress.

Based on the results of this pilot investigation on VMP35, an iron-free nutraceutical supplement, these researchers hypothesized that the co-incidence of anemia with diverse anaerobic-based pathologies has been wrongly conceptualized. The researchers discussed a new perspective on chronic anemia.

Anemia should now be recognized as an etiological antecedent, a primary underlying cause of all the chronic anaerobic pathologies and disorders mentioned above. This research team suggests that more accurate characterizations are needed to indicate two distinct types of anemia that are not caused by gene mutations. The first is ‘hemorrhagic anemia’, evidenced by either acute or chronic hemorrhaging resulting in a detectable and substantial loss of blood and its consequential alterations in all blood parameters, including reduced iron. The cause of the hemorrhage could be, for example, traumatic injury. The second type of anemia is CAS, which represents a wide variety of anemic pathologies resulting from a deficiency in alkaline buffers inducing the expenditure of histidine from heme groups, consequentially causing the anemic conditions. This defensive mechanism is apparently a precursor to numerous other anaerobic pathologies. The authors’ notion is that highly absorbable and comprehensive supplemental nutrient repletion from a VMP35 MNC will provide the buffers necessary to halt Hb expenditure, effectively pulling excess accumulated iron from other tissue storage depots, enabling rapid reconstitution of RBC Hb.

To test this hypothesis and based on our case studies, our team conducted a pilot clinical study and demonstrated that patent-pending VMP35 non-iron containing liquid supplement, enriched in diverse antioxidants, multivitamins, micronutrients, minerals, and standardized botanical phytonutrients and encapsulated in a novel multi-lamellar clustoidal Prodosomal absorption technology, rapidly improved morphological, hematological, and rheological properties of live human blood. Moreover, this study exhibited that enough nutritional and phytochemical resources were available from the VMP35 MNC to provide adequate buffering to restore intracellular RBC Hb within 5 min of intake that were sustained for at least 30 min post-intake. In addition, the properties of white blood cells including neutrophils significantly improved. Furthermore, the case study report is very encouraging, which strengthens the findings of Prodovite VMP35.

Taken together, VMP35, a non-iron-based nutraceutical formulation supplement, may serve as a novel therapeutic intervention in the restoration of Hb in RBCs and reverse progression of anemic pathologies considered IDA.

## Conflict of interests and funding

JRC is a highly respected board-certified practicing medical doctor whose clinical experience with the VMP35 confirmed the concept and hypothesis of this article and guided the writing of the manuscript. BWD, DB and SK contributed to the concept of the manuscript and are engaged with Victory Nutrition International, Inc., Lederach, PA, and ALM R&D, Oldsmar, FL, respectively. TA is an integral part of Veritas Health Inc., Woodbridge, ON, who organized the live cell imaging procedures. MB (Dr. Herbs LLC, Concord, CA) is an independent consultant, who compiled the data and routed the manuscript to all concerned.
